# Molecular Prevalence and Genotype Detection of *Babesia microti* in Multiple Wild Rodent Species and *Suncus murinus* From Subtropical Mountainous Regions of South‐Central Zhejiang, China

**DOI:** 10.1155/tbed/9501371

**Published:** 2026-10-21

**Authors:** Jie Xu, Jia-Qi Zhang, Jian-Feng Zhang, Yan Feng, Xuan Zhang, Wen-Jie Xu, Xiao-Xiao Wang, Ji-Min Sun, Wei Ruan

**Affiliations:** ^1^ School of Public Health Hangzhou Medical College Hangzhou Zhejiang China hznu.edu.cn; ^2^ Zhejiang Provincial Center for Disease Control and Prevention Hangzhou Zhejiang China cdc.zj.cn

**Keywords:** *Babesia microti*, Kobe genotype, molecular epidemiology, reservoir host, rodent, shrew, subtropical mountain, Zhejiang, zoonosis

## Abstract

Human babesiosis, an emerging zoonosis with ongoing geographic expansion, is primarily caused by *Babesia microti*, a tick‐borne apicomplexan parasite that resides within host erythrocytes. While small mammals serve as its recognized reservoir hosts, the relative contribution of sympatric wild rodents and shrews in subtropical mountain endemic areas of Eastern China remains poorly characterized. In South‐central Zhejiang, an endemic focus of human babesiosis, systematic data on local small‐mammal hosts remain limited. In this study, 630 small mammals, comprising six rodent species and the shrew *Suncus murinus*, were captured across six counties in Zhejiang Province of China. Host identity was confirmed by morphological examination and mitochondrial cytochrome b (*cytb*) barcoding. Whole‐blood DNA was screened for *B*. *microti* by TaqMan real‐time quantitative PCR (qPCR); positive specimens were further analyzed by nested PCR (NPCR) targeting the *18S rRNA* gene, followed by sequencing for genotype identification. The overall qPCR‐positive rate was 3.0% (19/630). Geographically, *B. microti* infections were detected in four out of six surveyed counties, with Jinyun County showing the highest prevalence of 6.9%, followed by Qingyuan (4.0%), Wencheng (3.2%), and Qingtian (3.0%); no positive samples were identified in the Yunhe and Jingning counties. Among the sampled species, *Niviventer lotipes* showed the highest prevalence (11.7%, 12/103), followed by *S. murinus* (3.8%, 2/52), *Alexandromys fortis* (3.7%, 3/81), *Apodemus agrarius* (1.0%, 1/99), and *Rattus norvegicus* (0.5%, 1/222). No positive results were detected in *R. tanezumi* (*n* = 62) or *Leopoldamys edwardsi* (*n* = 11). Fifteen of the 19 samples yielded positive amplification by NPCR and were successfully sequenced. All 15 sequenced isolates were assigned to the Kobe genotype and exhibited close genetic relatedness to previously reported local human‐derived and tick‐derived strains. The dominance of a single haplotype (*n* = 14) across host species and counties indicates genetically homogeneous circulation of the Kobe genotype within local small‐mammal communities, with host species rather than sampling county as the significant determinant of infection risk. These findings suggest that *N. lotipes* may be an important reservoir host in this region, and shrews may represent understudied secondary hosts, supporting the inclusion of both rodents and shrews in regional babesiosis surveillance.

## 1. Introduction

Human babesiosis is an emerging tick‐borne zoonosis caused by intraerythrocytic protozoan parasites of the genus *Babesia* (phylum Apicomplexa, order Piroplasmida) [1]. At least seven *Babesia* species have been reported to infect humans: *B. crassa*, *B. divergens*, *B. duncani*, *B. microti*, *B. motasi*, *B. odocoilei*, and *B. venatorum*. Among these, *B. microti* accounts for 95.3% of human babesiosis cases [2]. In humans, infection can range from mild influenza‐like illness to severe, life‐threatening hemolytic anemia, with immunocompromised individuals at particularly high risk [3]. Although rare secondary routes such as blood transfusion, perinatal transmission, and organ transplantation have been documented, tick bites remain the principal mode of transmission [3, 4].

Rodents and shrews live in close proximity to humans and may serve as a bridge among humans, domestic animals, and arthropod vectors. The enzootic cycle of *B. microti* involves interactions between ixodid tick vectors and these small‐mammal hosts [4]. Both rodents and shrews are considered important reservoir hosts of *B. microti*. Following infection, they can maintain persistent low‐level parasitemia, providing a sustained source of infection for feeding ticks [5]. Ticks acquire the parasite during blood meals on infected rodents and may subsequently transmit it to other susceptible vertebrate hosts via transstadial (but not transovarial) transmission. This cycle can support long‐term pathogen persistence in natural foci and may facilitate spillover into human populations [6]. Surveillance of *B. microti* carriage in wild small mammals is therefore valuable for characterizing local pathogen circulation and assessing public‐health exposure risks.

Conventional diagnostics for babesiosis include blood‐smear microscopy and serology [5]. Microscopy is labor‐intensive, requires skilled personnel, and may miss low‐parasitemia infections. Serology reflects cumulative past exposure and cannot reliably distinguish active infection from prior exposure [3]. Molecular assays, including real‐time quantitative PCR (qPCR) and 1*8S rRNA* gene‐targeted nested PCR (NPCR), offer improved sensitivity and specificity for detecting low‐grade parasitemia in wildlife samples and allow pathogen confirmation and genotyping by Sanger sequencing [1]. Molecular surveillance of wildlife reservoirs is important for investigating the *B. microti* enzootic ecology in endemic regions.

*Babesia microti* is the most frequently detected *Babesia* species in wild rodents and shrews, with infections reported across all inhabited continents except Oceania [2]. The genetic composition of circulating *B. microti* populations, however, differ considerably among regions and, consequently, among the host species involved. In North America, the white‐footed mouse (*Peromyscus leucopus*) serves as the principal reservoir host [7]. In Europe, the Munich strain, which is considered nonzoonotic, has been detected in bank voles [8, 9]. In East Asia, the Kobe genotype has been identified in multiple rodent genera in Japan and the Russian Far East [10, 11], whereas in mainland Southeast Asia, both U.S.‐type and Kobe‐type genotypes circulate among rodents and insectivores [12]. Recent continental surveys have also detected *B. microti* in African rodents [13].

In China, natural *B. microti* infections have been identified in wild rodents and shrews across Beijing, Henan, Xinjiang, Shanxi, Yunnan, Fujian, Guangxi, Zhejiang, and Taiwan, China [14–21]. The Kobe‐type predominates in China [20], while both Kobe‐ and Otsu‐types cocirculate in Yunnan Province [16, 22], and both Kobe‐type and U.S.‐type were identified in Beijing [14]. Nevertheless, *B. microti* carriage among wild rodents from areas with confirmed human babesiosis transmission remains poorly defined [23]. Autochthonous human *B. microti* babesiosis cases, including fatal infections among immunodeficient patients, have been documented in Southern and South‐central Zhejiang since 2012, yet baseline epidemiological data for local wild small‐mammal populations remain incomplete despite the presence of regional natural foci [20, 24]. Key knowledge gaps include infection prevalence among different host taxa, the relative reservoir contribution of individual host species, and the genetic diversity of circulating *B. microti* isolates.

We conducted systematic small‐mammal trapping and molecular detection of *B. microti* across six counties in South‐central Zhejiang during 2024–2025 to characterize the infection prevalence, spatial distribution, host associations, and genetic diversity of circulating strains. These findings provide baseline surveillance data for subtropical babesiosis natural foci and may inform the understanding of the multihost maintenance of *B. microti*.

## 2. Materials and Methods

### 2.1. Study Sites and Small‐Mammal Sampling

Field sampling was conducted from May 2024 to October 2025 across two cities and six counties in South‐central Zhejiang, Eastern China (Figure 1). This region has a subtropical monsoon climate, with a mean annual temperature of 16–19°C and an annual precipitation of 1400–1800 mm. The land cover is dominated by subtropical evergreen broad‐leaved forest, coniferous plantations, shrubland, riparian habitats, and rural residential areas interspersed with farmland.

**Figure 1:**
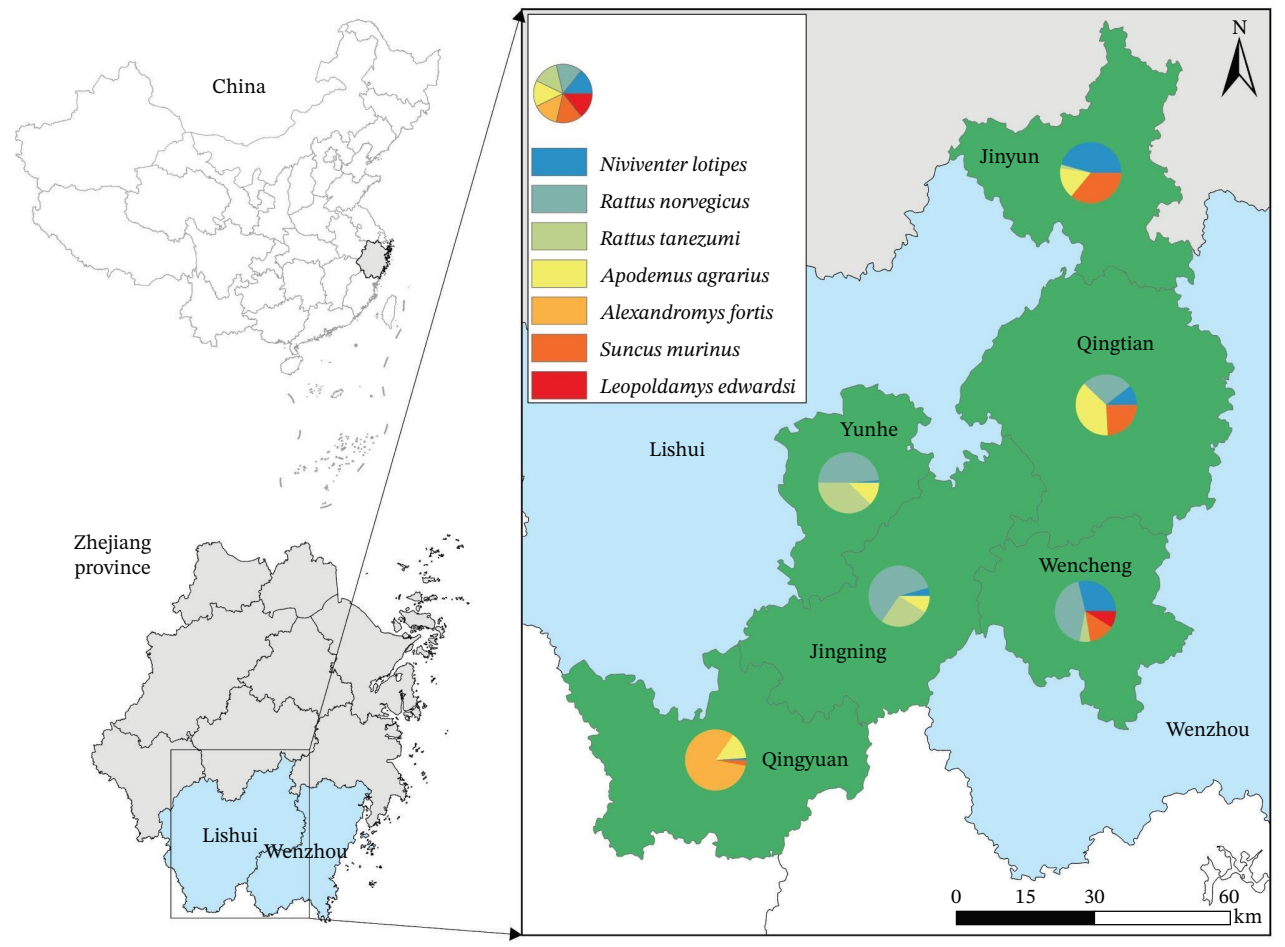
Location of the six sampling counties in South‐central Zhejiang Province, Eastern China. (Left) Map of China showing the location of Zhejiang Province highlighted. (Right) Enlarged administrative map displaying the six sampling counties (Yunhe, Qingyuan, Jingning, Jinyun, Qingtian, and Wencheng) in Lishui City and the adjacent part of Wenzhou City. Pie charts at each county depict the species composition of the small mammals captured, with species color‐coded as shown in the legend (*Niviventer lotipes*, *Rattus norvegicus*, *Rattus tanezumi*, *Apodemus agrarius*, *Alexandromys fortis*, *Suncus murinus*, *and Leopoldamys edwardsi*). The map was generated using ArcGIS 10.8 (Esri, Redlands, CA, USA). Scale bar, 60 km.

Small mammals were trapped overnight using live cage traps (28 cm × 13 cm × 10 cm) baited with peanut butter and dried fish. Traps were placed along linear transects at 50–100 traps per site per night and inspected at dawn. A total of 630 small mammals were collected: Yunhe (*n* = 82), Qingyuan (*n* = 99), Jingning (*n* = 90), Jinyun (*n* = 102), Qingtian (*n* = 132), and Wencheng (*n* = 125). Of these, 290 were captured in 2024 and 340 in 2025, with sampling conducted in both years within each county.

Animals were euthanized by cervical dislocation under anesthesia. The animal study was approved by the Ethics Review Board of the Zhejiang Provincial Center for Disease Control and Prevention (Approval Number 2024‐028‐01). The study was conducted in accordance with local legislation and institutional requirements. Whole‐blood samples were collected by cardiac puncture into EDTA microtubes, stored at −20°C in the field, and transferred to −80°C prior to DNA extraction. Species were initially identified using published morphological keys for Chinese mammals [25]. Specimens with ambiguous morphological features were confirmed by mitochondrial cytochrome b (*cytb*) barcoding, following the guidelines defined by Verma and Singh [26] for the primer design and PCR conditions.

### 2.2. DNA Extraction

Genomic DNA was extracted from 100 μL EDTA‐anticoagulated whole blood using the DNeasy Blood & Tissue Kit (Qiagen, Hilden, Germany) following the manufacturer’s protocol, with a final elution volume of 100 μL. DNA concentration and purity were assessed by NanoDrop 2000 spectrophotometry (Thermo Fisher Scientific, Waltham, MA, USA), with A260/A280 ratios of 1.7–2.0. Extracts were stored at −20°C until molecular screening.

### 2.3. Molecular Detection of *B. Microti*

*Babesia microti* screening was performed using TaqMan real‐time qPCR, which targeted an 85 bp partial fragment of the *18S rRNA* gene. The qPCR primer‐probe set included forward primer Bam‐F (5^′^‐TCA TTA AAT TYG CTT CCG AGC G‐3^′^), reverse primer Bam‐R (5^′^‐AAA TCC CGG AAA ATA GAA CCC C‐3^′^), and FAM‐BHQ1‐labeled probe Bam‐P (5^′^‐FAM‐TGA CTT GGC ATC TTC TGG ATT TGG TTC C‐BHQ1‐3^′^). The primer and probe sets, as well as the corresponding thermal cycling conditions, were adopted from our previous study by Zhang et al. [20]. The qPCR protocol included an initial denaturation step at 95°C for 5 min, followed by 40 amplification cycles (95°C for 10 s and 51°C for 35 s). Each PCR assay run was accompanied by validated positive specimen controls and nuclease‐free water blank controls to ensure assay reliability. To ensure reproducibility and minimize false‐positive results near the detection threshold, a Ct value of 35 was conservatively defined as the diagnostic cutoff; samples with Ct ≤ 35 were considered positive.

All qPCR‐positive samples were further subjected to NPCR to amplify a ~1600 bp fragment of the *B. microti 18S rRNA* gene. The outer primer pair BaC1F (5^′^‐GCC AGT AGT CAT ATG CTT GTG TTA‐3^′^)/BaC1R (5^′^‐CTC CTT CCT YTA AGT GAT AAG GTT CAC‐3^′^) and inner primer pair BaC2F (5^′^‐CCA TGC ATG TCT WAG TAY AAR CTT TTA‐3^′^)/BaC2R (5^′^‐CCT YTA AGT GAT AAG GTT CAC AAA ACTT‐3^′^) were used as previously reported by Wei et al. [14]. Both rounds of NPCR amplification were conducted under identical thermal conditions: initial denaturation at 95°C for 5 min, 20 cycles of denaturation (95°C for 30 s), annealing (60°C for 30 s), and extension (72°C for 90 s), followed by 17 cycles of adjusted annealing temperature (50°C for 30 s) with consistent denaturation and extension parameters, and a final extension step at 72°C for 10 min. Positive and no‐template negative controls were incorporated into each NPCR batch to rule out contamination. Amplified PCR products were resolved via electrophoresis on 1.5% agarose gels for band detection.

### 2.4. Sanger Sequencing and Genotype Assignment

All the NPCR products of the expected size were gel‐purified using the QIAquick Gel Extraction Kit (Qiagen) and then transferred to Beijing Tsingke Biotech Co., Ltd. (Hangzhou, China) for bidirectional Sanger sequencing. Chromatograms were inspected and assembled using SeqMan Pro (DNASTAR, Madison, WI, USA). Consensus sequences were compared against the NCBI GenBank nonredundant nucleotide database by BLASTn for species confirmation; sequences with ≥99% identity to published *B. microti* reference sequences were retained for downstream analysis. ClustalX Software Version 2.1.5.0 (http://clustal.org/, accessed on August 30, 2026) was used for multiple sequence alignment.

### 2.5. Genetic Diversity and Phylogenetic Analysis

DnaSP 6.12.03 software [27] was used for genetic diversity analysis. Nucleotide diversity (*π*) and the number of segregating sites per nucleotide were calculated to account for differences in the sample size and sequence length. Representative sequences capturing all unique haplotypes were used for analysis to avoid bias from redundant identical sequences.

Phylogenetic analysis was performed based on the partial *SSU rRNA* gene sequences (1567 bp) using MEGA 11 software. A neighbor‐joining (NJ) phylogenetic tree was constructed using the Kimura 2‐parameter nucleotide substitution model, and the reliability of tree topology was evaluated by 1000 bootstrap replicates. Representative reference sequences covering the major genotypes (Kobe, United States, Munich, and Otsu) of *B. microti* were included, with *Theileria annulata* used as the outgroup.

### 2.6. Statistical Analysis

Infection rates were calculated as the number of qPCR‐positive individuals divided by the total number tested, with 95% confidence intervals (CIs) derived using the Wilson score interval method. Differences in prevalence among host species, sampling counties, and years were assessed with Pearson’s chi‐square test (*χ* ^2^), and Fisher’s exact test was employed when expected cell frequencies were below five. Odds ratios (ORs) with 95% CIs were computed to compare infection risk across host species using the most abundant species (*R. norvegicus*) as the reference. All statistical analyses were performed using SPSS Version 22.0 (SPSS Inc., Chicago, IL, USA). A *p*‐value < 0.05 was considered statistically significant.

## 3. Results

### 3.1. Species Composition of Captured Small Mammals

A total of 630 small mammals were captured across six sampling counties during 2024–2025. Morphological and molecular identification revealed six rodent species (order Rodentia) and one shrew species (order Eulipotyphla, family Soricidae). *Rattus norvegicus* was the most abundant species (*n* = 222, 35.2%), followed by *Niviventer lotipes* (*n* = 103, 16.3%), *Apodemus agrarius* (*n* = 99, 15.7%), *Alexandromys fortis* (*n* = 81, 12.9%), *R. tanezumi* (*n* = 62, 9.8%), and *Leopoldamys edwardsi* (*n* = 11, 1.7%). The only shrew species was *Suncus murinus* (*n* = 52, 8.3%) (Table 1).

**Table 1:** Species composition of small mammals captured across six counties in South‐central Zhejiang, China.

County	NL	RN	AA	RT	SM	AF	LE	Total
Jinyun	47	37	17	1	0	0	0	102 (16.2)
Jingning	4	55	8	23	0	0	0	90 (14.3)
Qingtian	14	36	50	0	32	0	0	132 (21.0)
Qingyuan	1	0	14	0	3	81	0	99 (15.7)
Yunhe	1	40	10	31	0	0	0	82 (13.0)
Wencheng	36	54	0	7	17	0	11	125 (19.8)
Total	103 (16.3)	222 (35.2)	99 (15.7)	62 (9.8)	52 (8.3)	81 (12.9)	11 (1.7)	630 (100)

*Note:* Values in parentheses in the total row and total column indicate percentage of the overall 630 captures.

Abbreviations: AA, Apodemus agrarius; AF, Alexandromys fortis; LE, Leopoldamys edwardsi; NL, Niviventer lotipes; RN, Rattus norvegicus; RT, Rattus tanezumi; SM, Suncus murinus.

### 3.2. Overall Prevalence of *B. Microti*

Of 630 small‐mammal blood specimens screened by qPCR, 19 tested positive for *B. microti*, corresponding to an overall prevalence of 3.0% (95% CI: 1.9%–4.7%). Among these, 15 (78.9%) were successfully amplified by NPCR and confirmed by sequencing; the remaining four yielded no amplification products. The NPCR‐confirmed prevalence was 2.4% (15/630, 95% CI: 1.4%–4.0%). When stratified by sampling year, prevalence was 4.1% (12/290, 95% CI: 2.4%–7.1%) in 2024 and 2.1% (7/340, 95% CI: 1.0%–4.2%) in 2025; the difference was not statistically significant (*χ* ^2^ = 2.31, *df* = 1, and *p* = 0.128).

### 3.3. Stratified Prevalence by Host Species

*B. microti* infection was detected in four of six rodent species and in *S. murinus* (Table 2). *N. lotipes* showed the highest prevalence (11.7%, 12/103, 95% CI: 6.8%–19.3%), accounting for 63.2% (12/19) of all positive cases, followed by *S. murinus* (3.8%, 2/52, 95% CI: 1.1%–13.0%), *A. fortis* (3.7%, 3/81, 95% CI: 1.3%–10.3%), *A. agrarius* (1.0%, 1/99, 95% CI: 0.2%–5.5%), and *R. norvegicus* (0.5%, 1/222, 95% CI: 0.1%–2.5%). *R. tanezumi* (0/62, 95% CI: 0%–5.8%) and *L. edwardsi* (0/11, 95% CI: 0%–25.9%) tested negative. Interspecies differences in prevalence were significant (Fisher’s exact test, *p* < 0.001).

**Table 2:** *Babesia microti* infection status among small mammals stratified by host species, South‐central Zhejiang, China.

Host species	Order/Family	Number tested	Number qPCR+	Prevalence (95% CI)	Number NPCR+
*Alexandromys fortis*	Rodentia/Cricetidae	81	3	3.7 (1.3–10.3)	3
*Apodemus agrarius*	Rodentia/Muridae	99	1	1.0 (0.2–5.5)	0
*Leopoldamys edwardsi*	Rodentia/Muridae	11	0	0 (0–25.9)	0
*Niviventer lotipes*	Rodentia/Muridae	103	12	11.7 (6.8–19.3)	9
*Rattus norvegicus*	Rodentia/Muridae	222	1	0.5 (0.1–2.5)	1
*Rattus tanezumi*	Rodentia/Muridae	62	0	0 (0–5.8)	0
*Suncus murinus*	Eulipotyphla/Soricidae	52	2	3.8 (1.1–13.0)	2
Total	—	630	19	3.0 (1.9–4.7)	15

### 3.4. Stratified Prevalence by Sampling County

*B. microti* infection was detected in four of the six sampled counties (Table 3). Jinyun County showed the highest prevalence (6.9%, 7/102, 95% CI: 3.4%–13.5%), followed by Qingyuan (4.0%, 4/99, 95% CI: 1.6%–9.9%), Wencheng (3.2%, 4/125, 95% CI: 1.3%–7.9%), and Qingtian (3.0%, 4/132, 95% CI: 1.2%–7.5%). No positive samples were found in Yunhe (0/82) or Jingning (0/90). The prevalence of *B. microti* differed marginally across the six sampled counties (Fisher’s exact test, *p* = 0.049).

**Table 3:** *Babesia microti* infection prevalence among small mammals stratified by sampling county, South‐central Zhejiang, China.

County (city)	Number tested	Number qPCR+	Prevalence % (95% CI)	Positive host species (*n*)
Jingning (Lishui)	90	0	0 (0–4.1)	—
Jinyun (Lishui)	102	7	6.9 (3.4–13.5)	*N. lotipes* (6), *A. agrarius* (1)
Qingtian (Lishui)	132	4	3.0 (1.2–7.5)	*N. lotipes* (3), *R. norvegicus* (1)
Qingyuan (Lishui)	99	4	4.0 (1.6–9.9)	*A. fortis* (3), *S. murinus* (1)
Wencheng (Wenzhou)	125	4	3.2 (1.3–7.9)	*N. lotipes* (3), *S. murinus* (1)
Yunhe (Lishui)	82	0	0 (0–4.5)	—
Total	630	19	3.0 (1.9–4.7)	—

*N. lotipes* was the predominant positive host species, with infections detected in Jinyun (6/47, 12.8%), Qingtian (3/14, 21.4%), and Wencheng (3/36, 8.3%). *S. murinus* infections were detected in Qingyuan and Wencheng (one specimen each). *A. fortis* infections were detected exclusively in Qingyuan (three specimens). *A. agrarius* and *R. norvegicus* each yielded a single positive specimen, detected only in Jinyun and Qingtian, respectively.

### 3.5. Risk Factors Associated With *B. microti* Occurrence

Univariate and Firth‐penalized multivariate logistic regression were performed to identify factors associated with *B. microti* qPCR positivity (Table 4). For multivariate modeling, zero‐positive host species (*R. tanezumi* and *L. edwardsi*) and zero‐positive counties (Yunhe and Jingning) were excluded; sampling year was not included. *R. norvegicus* and Qingtian County served as reference groups for the host species and county, respectively. Univariate analysis showed that *N. lotipe*s had a significantly higher crude risk of infection (cOR = 20.17, 95% CI: 3.63–112.14, and *p* = 0.0006). After adjustment for host species and sampling county, *N. lotipes* remained the only independent risk factor (aOR = 11.48, 95% CI: 2.13–61.69, and *p* = 0.005). No other host species or sampling county was significantly associated with qPCR positivity.

**Table 4:** Univariate and Firth‐penalized multivariate logistic regression of factors associated with *B. microti* qPCR positivity.

Variable	Category	cOR (95% CI)	*P* (univariate)	aOR (95% CI)	*P* (multivariate)
Host species (vs. *R. norvegicus*)	*N. lotipes*	20.17 (3.63–112.14)	0.0006	11.48 (2.13–61.69)	0.005
	*A. fortis*	6.58 (0.95–45.66)	0.057	0.90 (0.06–14.67)	0.943
	*S. murinus*	7.31 (0.93–57.22)	0.058	3.55 (0.45–28.18)	0.232
	*A. agrarius*	2.25 (0.23–22.07)	0.487	1.03 (0.10–10.90)	0.979
	*R. tanezumi*	1.18 (0.05–29.99)	0.920	NA (excluded)	—
	*L. edwardsi*	6.42 (0.22–186.12)	0.279	NA (excluded)	—
Sampling county (vs. Qingtian)	Jinyun	2.24 (0.67–7.47)	0.188	0.97 (0.25–3.78)	0.967
	Qingyuan	1.35 (0.35–5.13)	0.664	3.22 (0.40–25.71)	0.269
	Wencheng	1.06 (0.28–4.02)	0.935	0.64 (0.16–2.61)	0.531
	Yunhe	0.17 (0.01–3.31)	0.244	NA (excluded)	—
	Jingning	0.16 (0.01–3.01)	0.220	NA (excluded)	—
Sampling year (vs. 2024)	2025	0.50 (0.20–1.26)	0.141	NA (not in model)	—

### 3.6. Molecular Characterization and Phylogenetic Analysis

High‐quality near‐full‐length *18S rRNA* gene sequences (1567 bp) were obtained from 15 of 19 qPCR‐positive specimens: nine from *N. lotipes*, three from *A. fortis*, two from *S. murinus*, and one from *R. norvegicus*. BLASTn searches confirmed that the 15 sequences shared ≥99.5% identity with the published *B. microti* Kobe‐type reference sequences in GenBank. Two haplotypes were identified: haplotype 1 (*n* = 14) and haplotype 2 (*n* = 1). The 14 haplotype 1 sequences (PZ868893) were completely identical and showed 100% nucleotide identity with the previously published human‐derived *B. microti* sequence (AB032434) isolated from Japanese patients, as well as with 31 additional sequences from rodents in Zhejiang, Beijing, and Taiwan, China (accession date: August 31, 2026). The single haplotype 2 sequence (PZ868907) had not been previously described; it differed from the 14 haplotype 1 sequences by one nucleotide and showed 99.94% identity with the reference sequence (AB032434). The overall nucleotide diversity was low (π = 0.000085).

Phylogenetic reconstruction based on the *18S rRNA* gene showed that the 15 study sequences grouped into a well‐supported monophyletic Kobe‐genotype clade (bootstrap = 99%), separated from U.S.‐type, Munich‐type, Otsu‐type, and *B. vulpes* clades (Figure 2). Within the Kobe‐genotype clade, the study sequences clustered closely with published Zhejiang human‐ and tick‐derived *B. microti* sequences (GenBank Accession Numbers MG674832, JQ609304, and PQ039648–PQ039675). The study sequences were genetically distinct from Kobe‐type isolates originating from Japan and other regions of China.

**Figure 2:**
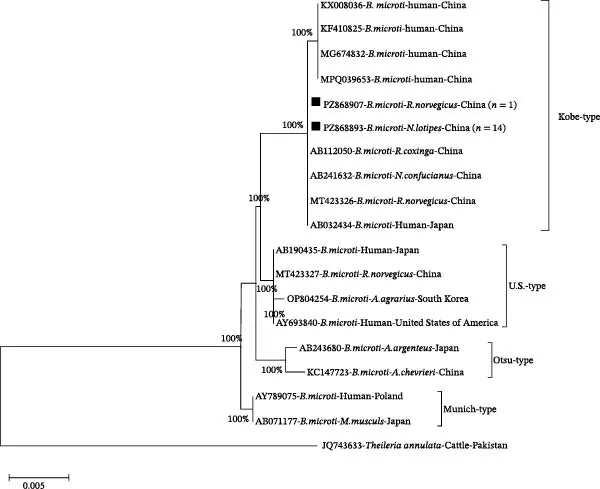
Neighbor‐joining phylogenetic tree based on the partial *18S rRNA* gene sequences (1567 bp) of *B. microti*, constructed using the Kimura 2‐parameter model with 1000 bootstrap replicates. The representative sequences obtained in this study are marked with black squares ⬛; reference sequences representing the Kobe, United States, Munich, and Otsu genotypes are included, with *Theileria annulata* used as the outgroup.

## 4. Discussion

The overall qPCR‐determined *B. microti* prevalence among small mammals in South‐central Zhejiang was 3.0% (19/630), comparable to reported values from Croatia (3.3%) [28], Lithuania (3.0%) [29], and Slovakia (3.2%) [30] and higher than those from France (0.17%) [31] and Turkey (0.6%) [32]. Moderately higher prevalences were reported from Southeast Asia (5.6%) [12] and Central Japan (4.6%) [11], whereas higher figures have been reported from the Republic of Korea (10.0%) [33], Gabon (10.3%) [34], and Chile (14.7%) [35].

Substantial geographic heterogeneity of *B. microti* prevalence exists across provincial‐scale rodent investigations in China. Our estimate was comparable to those from Fujian Province (3.96%) [19], Yunnan (2.4%–4.3% across two surveys) [16, 22], and a four‐province survey (4.17% for *Babesia* spp.) [36]. It was lower than reports from Southern Zhejiang (4.40%) [20], Central‐southern Shanxi (6.64%) [37], Henan (9.10%), Fujian (13.00%) [38], Taiwan (9.90%) [39], Southeastern Shanxi (10.71%) [17], and Beijing (12.10%) [14], while higher than results from the China–Myanmar border (1.80%) [40]. The observed variation, both between and within countries, emphasizes the need for region‐specific wildlife surveillance of babesiosis in China.

Multiple factors may account for the broad prevalence variation across published studies, including differences in diagnostic platforms, specimen matrix (whole blood versus spleen or liver tissues), sample size and host‐species composition, local tick‐vector abundance and competence, pathogen genotypes, habitat, elevation, and climate [2, 41]. Organ tissues often yield higher detection rates due to parasite sequestration during chronic infection, and qPCR has higher analytical sensitivity than conventional or NPCR and may detect low‐level residual pathogen DNA [42]. Heterogeneous trapping effort and interannual tick activity fluctuations further shape observed prevalence values [2]. Standardized sampling and diagnostic protocols would improve cross‐study comparability in future surveillance efforts.

Consistent with previous domestic findings, genus *Niviventer* exhibited an elevated infection risk. *N. lotipes* in the present study reached 11.7 % prevalence, over 20‐fold higher than that of *R. norvegicus* (0.5 %) and contributed nearly two‐thirds of all positive samples. This aligns with the high infection rates reported for *N. confucianus* in Shanxi and Beijing [14, 17, 37]. Host‐specific prevalence should be interpreted cautiously for species with limited sample sizes, as small sample numbers may bias susceptibility assessments.

All *B. microti* isolates successfully sequenced in this study belonged to the Kobe genotype, consistent with previous reports from Southern Zhejiang [20] and from Japan and the Russian Far East [10, 21]. The Kobe genotype is one of at least five recognized clades within the *B. microti* species complex, alongside the United States, Munich, Otsu, and *B. vulpes* types, each associated with distinct geographic distributions and tick‐vector species [2, 17]. Detection of the Kobe genotype in all 15 sequenced isolates spanning six counties and multiple host species indicates this is the dominant and potentially the only *B. microti* lineage circulating in South‐central Zhejiang small mammals. This pattern differs from regions such as Northeastern China (Heilongjiang, Inner Mongolia), where *B. venatorum*, *B. crassa*‐like organisms, and *B. microti* U.S.‐type strains cocirculate, reflecting the more diverse tick fauna (particularly *Ixodes persulcatus*) in those regions [43, 44]. Broader sampling across additional host species and geographic areas is needed to determine whether other lineages cocirculate at low levels in Zhejiang, China.

Among the 15 sequenced isolates, two *18S rRNA* haplotypes were identified: a dominant haplotype (haplotype 1, *n* = 14) that was 100% identical to previously published human‐derived *B. microti* sequences from Japan and a single‐nucleotide variant (haplotype 2, *n* = 1). The overall low nucleotide diversity (*π* = 0.000085) is consistent with a clonal or nearly clonal population structure with limited genetic recombination, as expected for a pathogen primarily transmitted by ixodid ticks with transstadial but not transovarial passage. The sharing of the dominant haplotype between small‐mammal isolates and previously reported human and tick isolates from the same region is compatible with a local enzootic cycle linking the wildlife reservoir to human infection [20]. Given the limited number of sequenced isolates (*n* = 15), larger sample sizes and additional genetic markers (e.g., β‐tubulin, CCTη) are needed to more fully characterize population structure and transmission dynamics [10]. Moreover, because haplotype identification relied on direct Sanger sequencing of bulk PCR products, mixed infections in which one haplotype was present at low abundance could not be detected; cloning‐based or deep‐sequencing approaches would be required to capture such coinfections.

The detection of *B. microti* DNA in *S. murinus* (2/52, 3.8%), with successful sequencing confirming the Kobe‐type identity, extends the known host range of this genotype in China. Shrews belong to the order Eulipotyphla and share habitat and tick exposure with rodents in forest and rural ecosystems [36]. The occurrence of identical or nearly identical *B. microti* haplotypes in both shrews and rodents suggest that shrews may be incidental hosts acquiring infection from the same tick population rather than maintaining a distinct transmission cycle. However, the possibility that shrews contribute to enzootic maintenance cannot be excluded, particularly given the substantial trapping numbers of *S. murinus* in certain habitats (e.g., 32 individuals captured in Qingtian County alone). High *B. microti* infection rates have been documented among shrew species in other geographic regions (30.3% in *Sorex araneus* in the United Kingdom) [45], further highlighting the need to clarify the role of shrews in the *B. microti* transmission ecology. Experimental infection studies and xenodiagnosis would be required to formally assess the reservoir competence of *S. murinus* for *B. microti*.

### 4.1. Limitations

Several limitations of this study should be noted. First, sampling was restricted to six counties in South‐central Zhejiang, and the findings may not be generalizable to other regions. The absence of *B. microti* positives in Yunhe and Jingning may reflect limited sampling in specific microhabitats rather than true absence of transmission; expanded surveillance across additional counties, habitat types, and elevation gradients is warranted. Second, detection relied exclusively on nucleic acid amplification from whole‐blood specimens. qPCR positivity cannot distinguish active transmissible parasitemia from transient or residual DNA, and blood‐only sampling may underestimate the prevalence during chronic infection when parasites sequester in spleen and liver tissues. The four qPCR‐positive/NPCR‐negative samples may represent very low‐level infections or false‐positive signals. Combining serological assays (IFA or ELISA) with PCR testing of organ tissues, such as the spleen and liver, would improve assessment of infection status and cumulative exposure. Third, concurrent sampling of ticks and human populations was not performed, limiting direct inference of transmission dynamics and zoonotic risk. Although *Ixodes granulatus* has been implicated as the primary vector in this region [20], vector composition and infection rates may vary temporally and spatially. Integrated One Health surveillance incorporating hosts, vectors, and humans would clarify local transmission dynamics. Fourth, sample sizes were small for certain host species (e.g., *L. edwardsi*, *n* = 11), yielding imprecise prevalence estimates with wide CIs. Future studies with larger sample sizes, multitissue analysis, and serology are needed to evaluate the epidemiological role of each host species more rigorously; experimental infection studies and xenodiagnosis are recommended as follow‐up investigations to formally confirm reservoir competence.

## 5. Conclusion

This study provides a systematic molecular epidemiological investigation of *B. microti* among wild rodents and shrews in South‐central Zhejiang Province, China. The results confirm active enzootic circulation of the *B. microti* Kobe genotype at an overall prevalence of 3.0%, with significant variation across host species. *N. lotipes* is identified as the predominant reservoir host, exhibiting a prevalence of 11.7% and accounting for nearly two‐thirds of all positive specimens. The detection of *B. microti* in *S. murinus*, with molecular confirmation of the Kobe genotype, suggests that shrews may represent previously overlooked secondary hosts within the local transmission cycle. Phylogenetic and haplotype analyses demonstrate that small‐mammal‐derived isolates are genetically closely related to previously reported human and tick isolates from the same region, reinforcing the role of small mammals as the primary reservoir source for human *B. microti* infection. These findings underscore the importance of incorporating both rodents and sympatric insectivores into babesiosis surveillance programs and highlight the need for integrated One Health approaches encompassing humans, ticks, and diverse mammalian hosts to fully characterize the enzootic ecology and zoonotic risk of *B. microti* in subtropical China.

## Author Contributions

**Jie Xu**, **Jia-Qi Zhang**, **Jian-Feng Zhang, and Wen-Jie Xu**: writing – original draft, investigation, formal analysis, software. **Yan Feng**: writing – original draft, investigation, formal analysis. **Xuan Zhang**: writing – original draft, sample collection, DNA extraction. **Xiao-Xiao Wang and Ji-Min Sun**: writing – review and editing, supervision, resources, conceptualization. **Wei Ruan**: writing – review and editing, writing – original draft, validation, software, resources, project administration, investigation, funding acquisition, formal analysis.

## Funding

This study was supported by the Zhejiang Science and Technology Plan for Disease Prevention and Control (Grant 2026JKY079).

## Ethics Statement

All procedures involving animals were reviewed and approved by the Institutional Animal Care and Use Committee (wildlife‐investigation ethics committee) of the Zhejiang Provincial Center for Disease Control and Prevention (Approval Number 2024‐028‐01). Trapping, handling, and euthanasia of small mammals were performed in accordance with national and institutional guidelines for the care and use of laboratory animals and wildlife.

## Consent

The authors have nothing to report.

## Conflicts of Interest

The authors declare no conflicts of interest.

## Data Availability

The *18S rRNA* gene sequences generated in this study have been deposited in the NCBI GenBank database (Accession Numbers PZ868893–PZ868924). All other data supporting the findings of this study are available from the corresponding authors upon reasonable request.
